# Bubbly flow prediction with randomized neural cells artificial learning and fuzzy systems based on * k–ε* turbulence and Eulerian model data set

**DOI:** 10.1038/s41598-020-70672-0

**Published:** 2020-08-14

**Authors:** Meisam Babanezhad, Mahboubeh Pishnamazi, Azam Marjani, Saeed Shirazian

**Affiliations:** 1 Institute of Research and Development, Duy Tan University, Da Nang, 550000 Vietnam; 2 Faculty of Electrical – Electronic Engineering, Duy Tan University, Da Nang, 550000 Vietnam; 3 Department of Chemical Sciences, Bernal Institute, University of Limerick, Limerick, Ireland; 4 Department of Chemistry, Islamic Azad University, Arak, Iran; 5 Department for Management of Science and Technology Development, Ton Duc Thang University, Ho Chi Minh City, Vietnam; 6 Faculty of Applied Sciences, Ton Duc Thang University, Ho Chi Minh City, Vietnam

**Keywords:** Engineering, Mathematics and computing

## Abstract

Computing gas and liquid interactions based on interfacial force models require a proper turbulence model that accurately resolve the turbulent scales such as turbulence kinetic energy and turbulence dissipation rate with cheap computational resources. The *k −* *ε* turbulence model can be a good turbulence predictive tool to simulate velocity components in different phases and approximately picture the turbulence eddy structure. However, even this average turbulence method can be expensive for very large domains of calculation, particularly when the number of phases and spices increases in the multi-size structure Eulerian approach. In this study, with the ability of artificial learning, we accelerate the simulation of gas and liquid interaction in the bubble column reactor. The artificial learning method is based on adaptive neuro-fuzzy inference system (ANFIS) method, which is a combination of neural cells and fuzzy structure for making decision or prediction. The learning method is specifically used in a cartesian coordinate such as Eulerian approach, while for the prediction process, the polar coordinate is applied on a fully meshless domain of calculations. During learning process all information at computing nodes is randomly chosen to remove natural pattern learning behavior of neural network cells. In addition, different $r$ and $\theta$ are used to test the ability of the learning stage during prediction. The results indicate that there is great agreement between ANFIS and turbulence modeling of bubbly flow within the Eulerian framework. ANFIS method shows that neural cells can grow in the domain to provide high-resolution results and they are not limited to the movement or deformation of source points such as Eulerian method. In addition, this study shows that mapping between two different geometrical structures is possible with the ANFIS method due to the meshless behavior of this algorithm. The meshless behavior causes the stability of the machine learning method, which is independent of CFD boundary conditions.

## Introduction

Bubble column reactors are multiphase domains to provide physical and chemical interaction between gas, liquid, and solid phases^1–5^. This physical interaction between two distinct phases can be observed in bubbly flow in the bubble column reactor. The sparging of gas into the continuous phase results in forming gas bubbles in continuous phase (e.g. liquid phase). This formation of dispersed into the continuous phase can be defined as a bubbly flow. However, in very low flow rates, bubbles are uniform and spherical shape, and by increasing the gas flow, bubbles start merging or breaking into a number of small bubbles. To design and optimize reactors and bubbly flow in the domain, understanding bubble column reactor hydrodynamics such as gas hold-up, gas and liquid flow pattern, bubble size and shape specification, and turbulence parameters are important factors in different applications and industries^6^. For local observation of these parameters, advanced mathematical models, numerical methods and proper discretization algorithms enable us to fully learn the behavior of the multiphase flow in reactors. Different types of CFD and mathematical modeling have been presented by researchers to analyze the hydrodynamics of bubble column reactors^2,7–14^. Among all numerical models, Euler–Euler approach based on a fix coordinate can compute movement of dispersed phase in the continuous phase (matrix phase) and the interaction between two phases by interfacial force models such as drag, lift, turbulence dispersion and wall lubrication force schemes. Beside the interfacial force model, turbulence modeling can participate in computing velocity components and eddy sizes, and it can compute different phases and the interaction between eddies due to coalescence and break-up of gas bubbles. This filtration mechanism can provide an accurate prediction of turbulence flow behind the multiphase behavior in the bubble column reactor^2,13,15^.

As the single size Euler–Euler approach is not enough to simulate range of bubble size and shape in the reactor, the multi-size Eulerian method or population balance method plus Eulerian framework must be replaced with a single approach to fully resolve the distribution of bubble size and accurate CFD prediction^16^. The calculation of exact bubble size distribution and the interaction between each bubble with population balance method is very expensive and requires supercomputer or cloud computing which is an on-demand cluster resource. These advanced computer resources can provide on-demand availability for big data storage and computer power without management of cluster maintenance. Cloud computing system allows industries to avoid or reduce up-front IT infrastructure costs for computing fluid flow in the domain, but it can be very expensive for individual researchers and academic environment. To avoid financial issues when we face huge computational calculations, particularly computing multiphase flow interaction and particle/bubble size distribution in the reactor, we need to redesign our computational process. One way to change computational time is to develop new computational techniques such as optimized parallel computing codes, new method for calculation of Navier–Stokes equations and machine learning methods. About a decade ago, machine learning methods were used as interpolation within the physical process. We cannot always use machine learning for prediction of the process, as machine learning fully depends on training of data set. By evolution of machine learning methods particularly in prediction of face recognition or physical process^17–19^, some researchers used these algorithms to train local values of CFD and then predict that local values for “*not training conditions*” in a very short computational time^20,21^. They also show that after learning process, the prediction period is very small, and there is no limitation of prediction of non-training conditions. Machine learning not only can be used for prediction of local values, but also for store big data set in a short memory and categorize many operational conditions in a fuzzy logic structure.

As these algorithms are growing, we need to find out the best way to verify training process for local data set. One way that we can use during training is to randomly shuffle big data set and then let the training method learn the data set. In this case, we can stop learning of machine learning from pattern of data, and it is only based on location of each node^22,23^. In this study, we compute the interaction between dispersed and continuous phase and velocity of each component in a single size Eulerian framework beside RANS turbulence model. ANFIS method is coupled with CFD results to mimic the flow in the column. Neural network is used in the training process, while the randomization method is used to mix all data set and then the fuzzy system used all information of cells for the purpose of decision and prediction step. In particular, the ANFIS method is used to predict the amount of gas fraction for each element of the bubble column reactor at a specific location in three dimensions. This method is also used for the AI mesh refinement process for the higher resolution of gas fraction in the reactor.

## Method

### CFD

**Figure 1:**
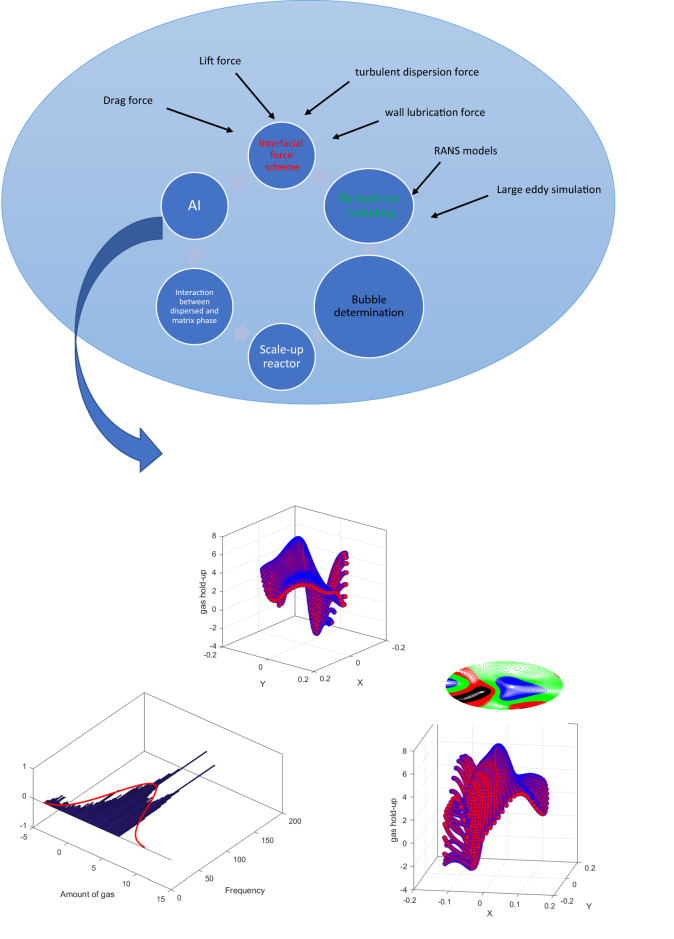
Design and forcing scheme for simulation of multiphase bubble column reactor by CFD and AI.

There are many studies to present high numerical resolution of local hydrodynamic parameters in multiphase bubble column reactors. The Eulerian approach or called multi-fluid model is frequency used to solve matrix (continuous) phase and dispersed phase at fixed coordinate. To solve bubbly flow and turbulence behavior beside each bubble, this method is usually devoted beside wide range of turbulent models, e.g. Reynolds stress model, k–ɛ model, explicit algebraic stress model, and large eddy simulation for higher numerical resolutions. A better understanding of transport phenomena and the interaction between phases beside the proper turbulence technique and efficient parallel algorithms enable us to achieve higher numerical resolutions multi-dispersed phase systems. Solving bubbly flow requires resolving interfacial force model and turbulence modeling. In case of interfacial force model based on behavior of flow in the column, different coupling forces can be used such as drag, lift, wall lubrication and turbulent dispersion force. The turbulence model can also calculate velocity components for each phase. Other factors during design of a numerical method for bubbly flow are size of dispersed phase, scale-up factors (size of the reactor) and other physical or chemical interaction between different phases. Figure 1 represents the connection between forcing schemes and AI modeling beside the scaling reactor from small scales to large scales. Figure 1 also shows that for modeling of two-phase flow, different interfacial force models are required, such as drag and lift forces. The main calculation of these forces is based on momentum equations. In addition to the calculation of the momentum term, the velocity components can be solved in an appropriate turbulence model. Additionally, the scale-up information for bubbly flow can be beneficial for build-up the model. After gathering all information, they can translate into the AI framework for faster presentation and categorization.

In this method, the gas and liquid phases are presented as continuous, fully interpenetrating continua, coupled by an interaction term. The governing equations of continuity as well as momentum for each phase is presented^6,24^:

1 $$ \frac{{\partial \left( {\rho_{k} \epsilon_{k} } \right)}}{\partial t} + \nabla \left( {\left( {\rho_{k} \epsilon_{k} u_{k} } \right)} \right) = 0 $$

In this equation, $$k $$ represents each phase, gas/liquid, and by adding phases into the continuity calculation, more interaction is added. $$\rho$$ describes the density of each phase and $$t$$ defines the transient calculation for phases.

To solve the interaction between phases, the momentum must be calculated. The momentum term is written as^13,24^:

2 $$ \frac{{\partial \left( {\rho_{k} \epsilon_{k} } \right)}}{\partial t} + \nabla \left( {\left( {\rho_{k} \epsilon_{k} u_{k} } \right)} \right) = - \nabla \left( {\epsilon_{{\text{k}}}\uptau _{{\text{k}}} } \right) - \epsilon_{{\text{k}}} \nabla p + \epsilon_{k} \rho_{k} g + M_{i,k} $$

The term $M_{i,k}$ presents calculation between each phase and in this study, the drag term is used as a predominant coupling mechanism for the interfacial force between two phases. As the interaction between spherical bubbles is low and the rate of coalescence and break-up is very small, the constant drag coefficient can be used in the modeling. In the momentum equation $\tau$ and $g$ represents shear and gravity, respectively.

To resolve the velocity of each component and turbulence eddy in the column, the *k −* *ε* turbulence model is used. In this model, the turbulent kinetic energy is presented as^13^:

3 $$ \frac{{\partial \left( {\rho_{K} } \right)}}{\partial t} + \frac{{\partial \left( {\rho_{K} U_{i} } \right)}}{{\partial x_{i} }} = \frac{\partial }{{\partial x_{i} }}\left[ {\frac{\mu t}{{\sigma_{k} }}\frac{\partial K}{{\partial x_{i} }} } \right] + 2\mu_{t} E_{ij} E_{ij} - \rho \epsilon $$

4 $$ \frac{{\partial \left( {\rho \epsilon } \right)}}{\partial t} + \frac{{\partial \left( {\rho_{\epsilon } U_{i} } \right)}}{{\partial x_{i} }} = \frac{\partial }{{\partial x_{i} }}\left[ {\frac{\mu t}{{\sigma_{\epsilon } }}\frac{\partial K}{{\partial x_{i} }} } \right] + C_{1\epsilon } \frac{\epsilon }{K}2\mu_{t} E_{ij} E_{ij} - C_{2\epsilon } \frac{{\rho \epsilon^{2} }}{K} $$

where $U_{i}$ describes the velocity of one phase in a different direction, $E_{i}$ represents deformation rate and eddy viscosity in each phase, and can be described as $\mu_{t}$. The detailed description of turbulence models, particularly *k −* *ε* can be found specifically in Tabib et al.^13^. They showed the impact of this model on accuracy of two phase results.

### CFD and AI grid

The non-structure grid is generated throughout the domain based on the high accuracy of the CFD method for the prediction of gas fraction and liquid pattern in the center of the domain and wall zones. In addition to CFD mesh, the Artificial Intelligence (AI) elements are generated individually in the programming function. This element structure contains millions of numerical structure cells, and they are not sensitive to the numerical convergence or numerical instability. In addition, the AI grid structure enables us to optimally post-process AI data as the location of each AI cells can be identified mathematically.

### Discretization

The finite volume method (FVM) is employed here for discretization of the Navier–Stokes equations in the form of algebraic equations and numerical structure. In the form of mathematical structure, volume integrals in a complex Navier–Stokes equation, which contain a divergence term, are translated to surface integrals using the divergence theorem. The numerical method is validated with previous studies for gas fraction and liquid pattern in the domain, near spargers, walls, and bulk regions.

### Geometry and the computational space

**Figure 2:**
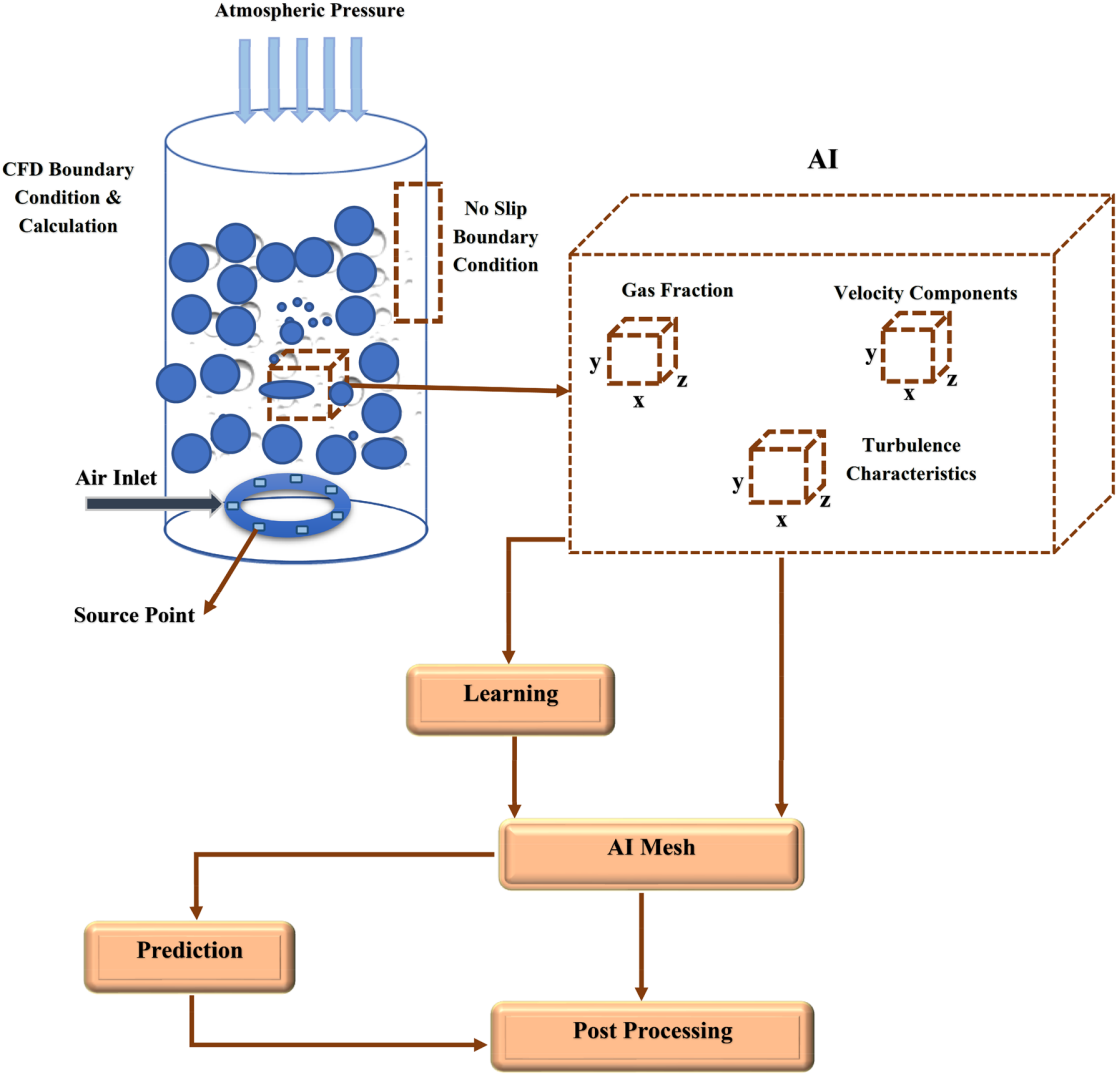
Schematic representation of CFD boundary conditions and AI flow chart as well as input parameters for AI framework.

The domain of liquid is designed in a three-dimensional cylindrical shape. The gas injected into this domain by source point spargers at the bottom of the domain. These source points continuously transfer gas into the domain of liquid and generate two-phase flow in the domain. Each source point sparger is 0.5 mm, and 20 source points are generated at the bottom of the domain in a ring shape. The operating pressure and temperature for this domain is based on average room conditions and atmospheric pressure. Figure 2 represents the boundary conditions near bubble column reactor wall, pressure outlet at the outlet and source point as input boundary conditions. In addition, this figure shows the selection of CFD data by AI framework.

### Adaptive neuro-fuzzy inference system (ANFIS)

#### Data selection

The position of each CFD elements is selected as input parameters of the method, while the gas fraction for each computing element is considered as an output parameter of the ANFIS method. All data are selected in the post-processing step, and after CFD calculation and solving the Navier–Stokes equation. The volume fraction of each element also represents the gas hold-up at that local point. The overall gas hold-up in the system is the average value of all computing nodes. In addition, the collection of CFD data set for local points are described in the schematic Fig. 2.

To train local CFD data, the neural network is used throughout the domain and for the prediction part, the fuzzy logic structure is coupled with a neural network framework. All hydrodynamic parameters are trained in fixed Eulerian Cartesian framework $\left\{ {X_{i} , Y_{i} , Z_{i} } \right\}$ and then represent in polar coordinates as:

5 $$ A_{i,\alpha }^{1} = X_{i }^{{\text{h}}} {\cos}\left( {\uptheta } \right) $$

6 $$ A_{i,\alpha }^{2} = Y_{i }^{{\text{h}}} {\sin}\left( {\uptheta } \right) $$

$$A_{i,\alpha }^{1} $$ and $$A_{i,\alpha }^{2}$$ represent prediction values in different polar coordinate. $$h $$ presents the level of the column that can be randomly selected in the training method.

**Figure 3:**
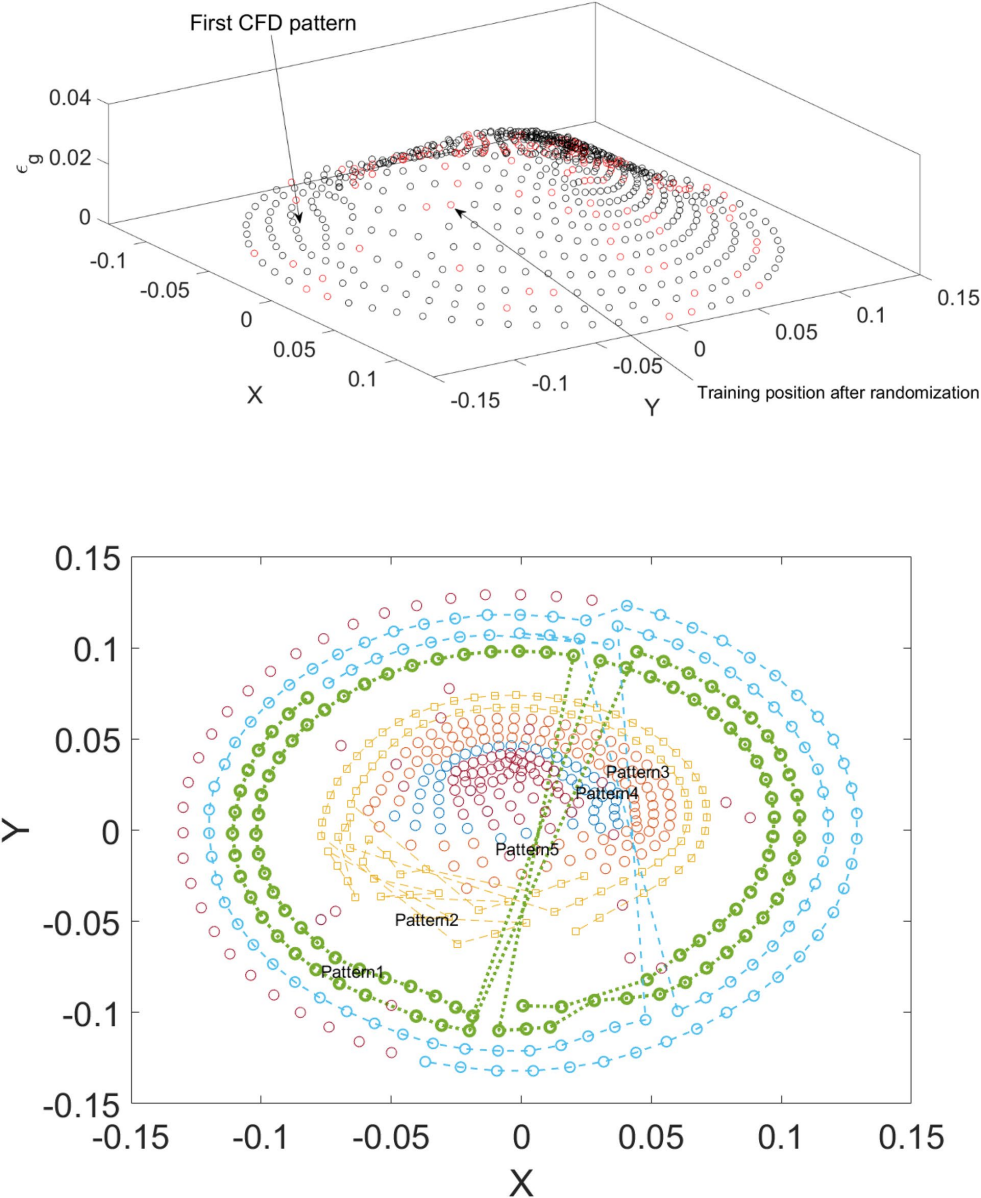
Amount of gas for each computing cell at the top surface of the column based on random selection of cells and connectivity between data set pattern. Here five different pattern data set is presented.

For the training data set we used *randperm* function to determine the amount of gas for each cell. In this case, we destroyed the pattern learning in ANFIS method and force this algorithm to learn the data set based on a fully random framework. The randomization is implemented throughout the fixed Cartesian framework in X and Y directions at different heights of the column (Fig. 3):

7 $$ R{ }\left\{ {X_{i}^{h} , Y_{i}^{h} } \right\} $$

**Figure 4:**
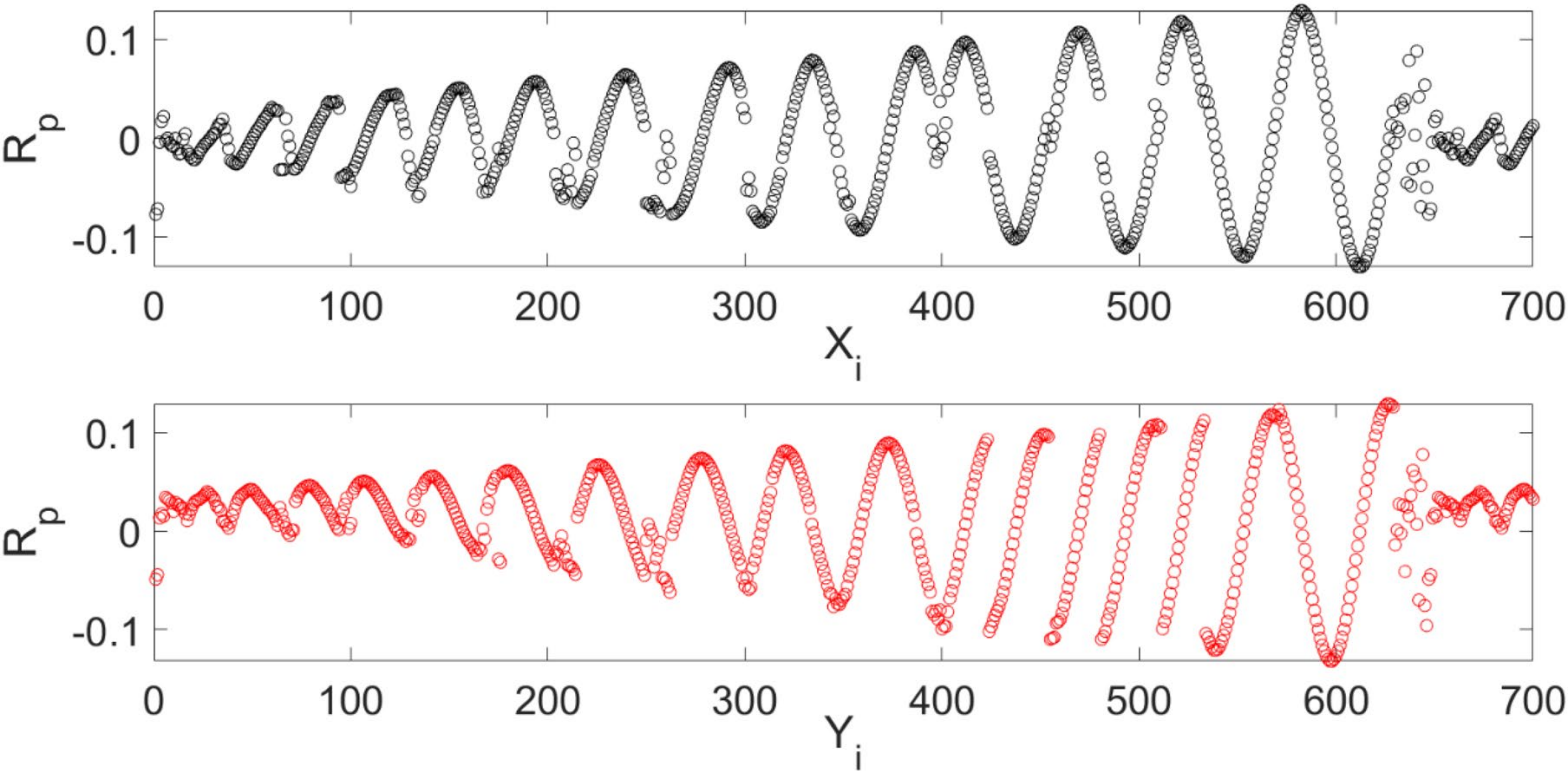
Random position of each node computing position for 700 selection nodes.

Figure 4 shows the random position of each node for 700 selection nodes. This way of training of computing nodes from random position enables us to stop the algorithm to get the rhythm of data set and only judge about the amount of gas for non-dimension points. The main idea of using a random framework to select the CFD data set is also about repeatability of the model with regards to the prediction of bubble column hydrodynamics. In this case, the machine learning method can be used many times, regardless of data selection^6^.

Based on *AND* law, the first feedback from the training step multiplies. The function _*j*_th rule can be described as:

8 $$ \omega_{j} = \alpha_{{a_{j} }} \left( {input1} \right)\beta_{{b_{j} }} \left( {input2} \right)\eta_{{c_{j} }} \left( {input3} \right) $$

where $\omega_{j}$ represents output feedback and $\alpha_{{a_{j} }} , \beta_{{b_{j} ,}} ,\eta_{{c_{j} }}$ represent input of learning feedback.

In another level of learning weight fraction of each neural network layer is defined as^6^:

9 $$ \overline{\omega }_{j} = \frac{{\upomega _{{\text{j}}} }}{{\sum \omega_{j} }} $$

where $\overline{\omega }_{j}$ shows the normalized weighting factor.

## Numerical implementation

In this study, the Navier–Stokes equations are resolved with a single size Eulerian method to compute multiphase gas–liquid interaction inside the reactor. The sparging gas in the reactor is modeled by mass source point calculation exactly at the sparger nodes in the reactor. At the top of the reactor, the degassing boundary conditions are used to avoid moving liquid from the top surface and removing gas from the top. The body of the reactor is no-slip boundary condition, while for gas–phase the free slip boundary condition is used in the model. The single size Eulerian model is used to reduce the computational costs, as the multi-size methods with population balance calculations require high computational specifications and high-performance clusters^25^.

As the behavior of the bubbly flow is turbulence multiphase flow, we use RANS turbulence modeling beside the calculations between gas and liquid phase to avoid uncertainty and risk during the design process for different flow conditions. The *k −* *ε* is used as a turbulence model to average phase velocity components and mean bubbly flow characteristics in turbulence flow conditions, and this model can provide standard criteria for evaluating turbulence kinetic energy and turbulence dissipation rate. This turbulence model can compute two main transported variables called turbulent kinetic energy and turbulence dissipation rate in the bubbly flow^26^.

After calculation of turbulence scales, the CFD results are used for training mode of ANFIS method. In this case, the three-dimensional bubble column reactor is calculated in the CFD framework and then the results are used for artificial training. In this study, we concentrate on the procedure of the training and how we can deal with a big-data set of multiphase flow. During training of the method, CFD nodes in different dimensions are trained as inputs and the flow characteristics are trained as outputs.

## Results and discussion

**Figure 5:**
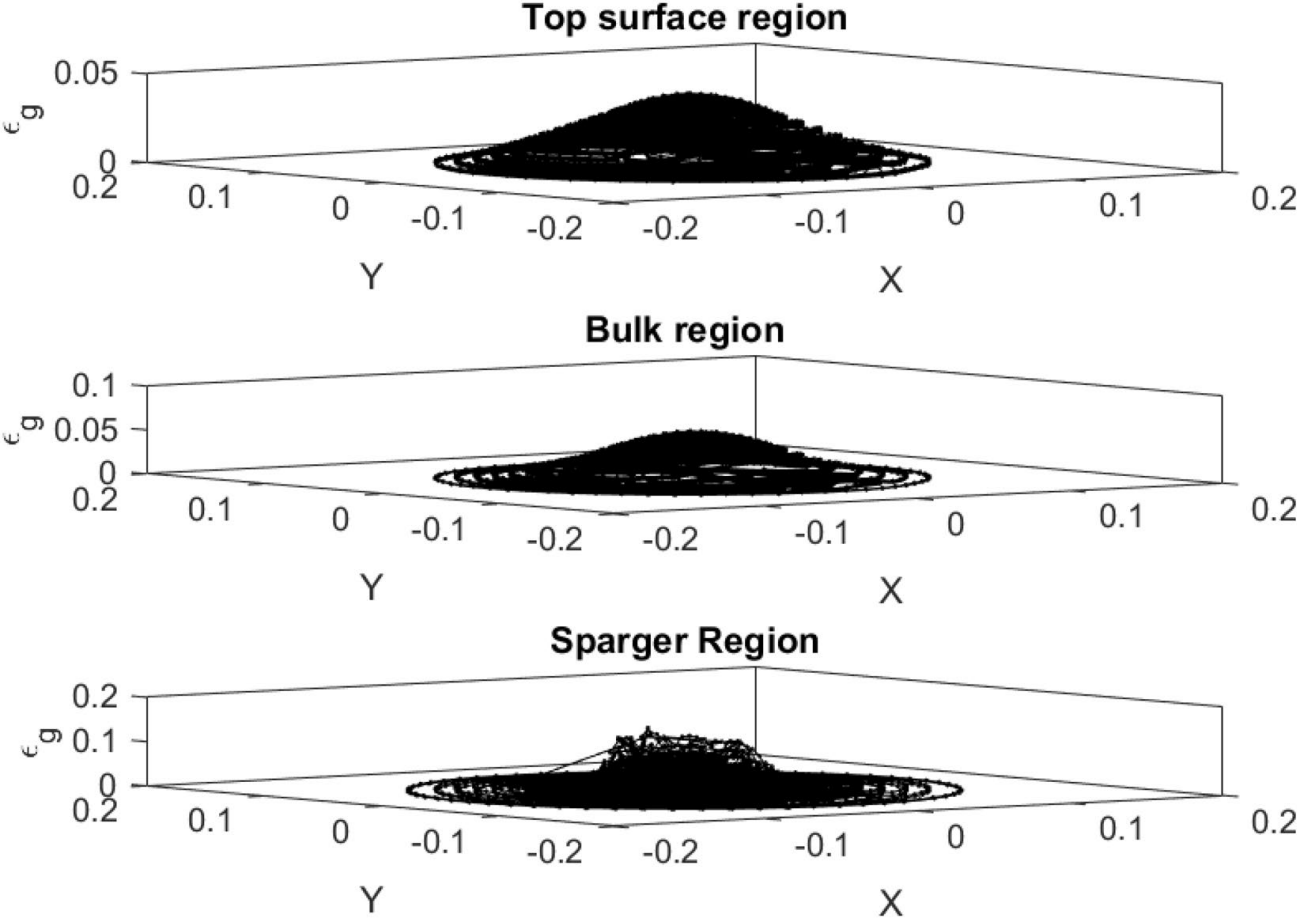
Amount of gas hold-up at X and Y computing points for the surface, bulk region and sparger region of the reactor.

Figure 5 illustrates the amount of gas hold-up at different levels of the column. Near the sparger region the gas fraction is higher than other parts of the reactor, while increasing the level of the reactor gas hold-up decreases. In addition, the gas profile is uniformly distributed throughout the column on the surface of the reactor due to full dispersion of bubbles across the column.

**Figure 6:**
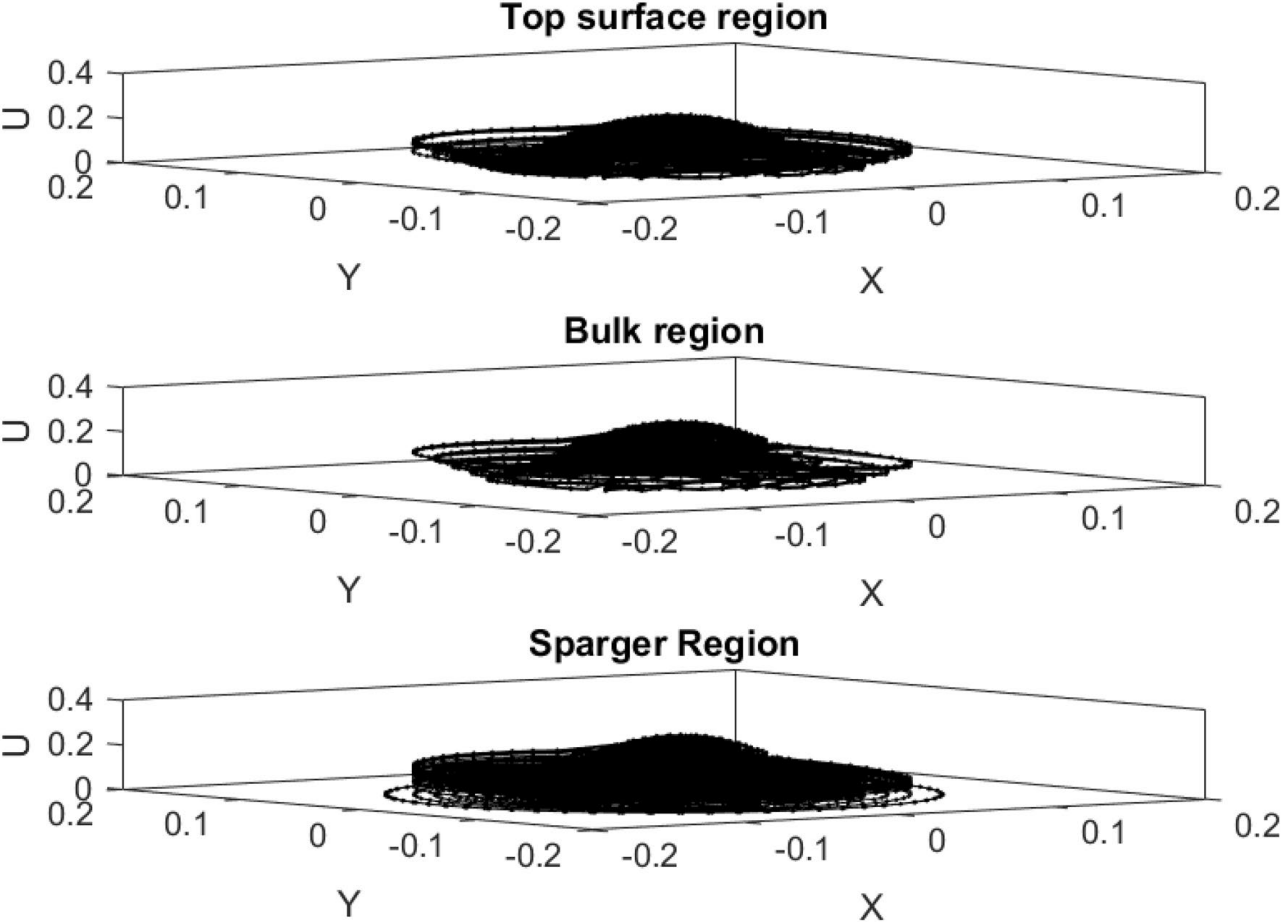
Velocity of liquid component $U = \sqrt {U_{x}^{2} + U_{y}^{2} + U_{z}^{2} )}$ at $X$ and $Y$ computing points for top surface, bulk region and sparger region of the reactor.

**Figure 7:**
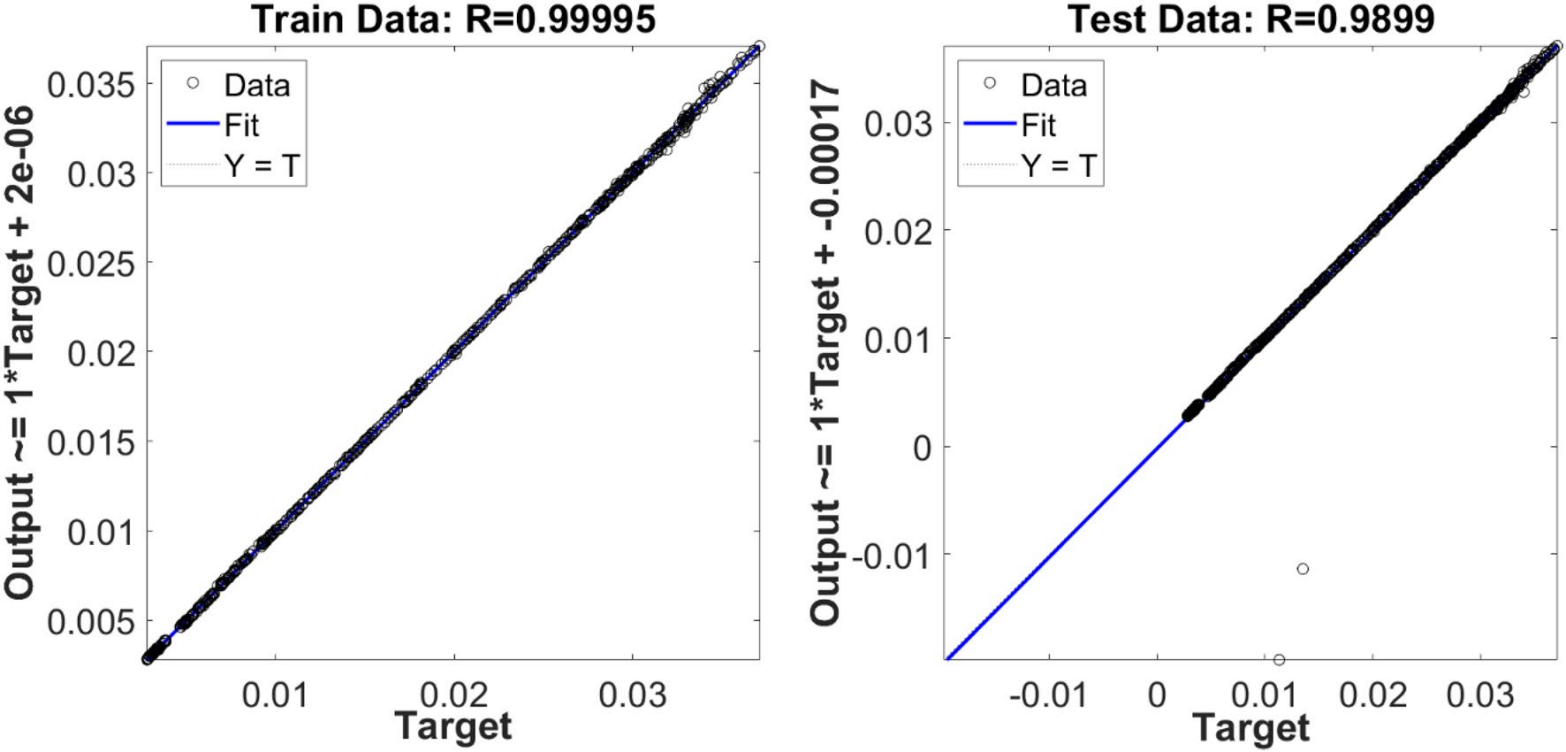
Training and testing accuracy results.

**Figure 8:**
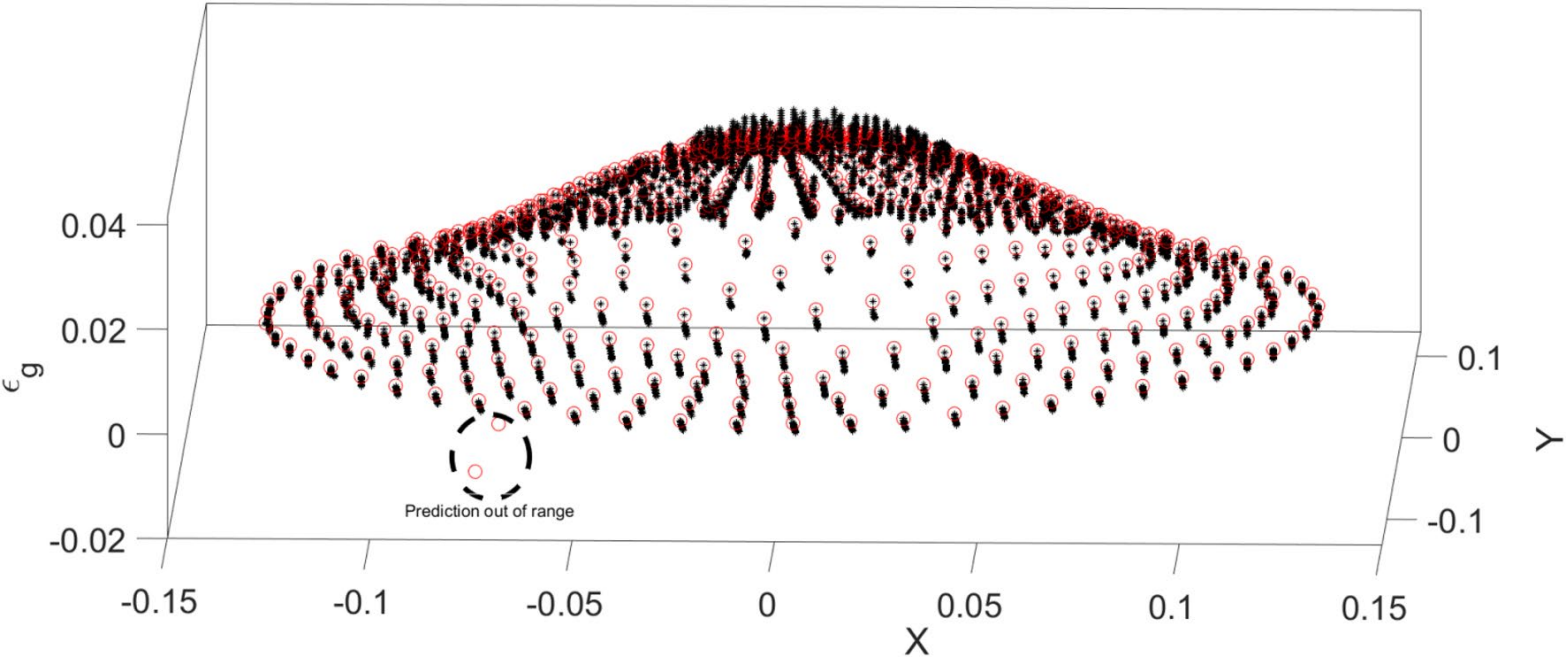
Prediction of gas hold-up with ANFIS method and compared with CFD results at the top surface of the reactor.

Figure 6 also shows the liquid velocity across the column for a different level of the reactor. Near the sparger region, the liquid velocity is high particularly near the sparger source point. By increasing the height of the reactor, the liquid is more uniformly distributed in the column. For training of CFD data, 70% of full data is selected during training and then, we evaluated the accuracy of data in the training. After learning process, 30% of data is tested and evaluated with CFD results. For evaluation of training method, we compared training data set with predicted values with ANFIS method, while for testing method, we also compared all data set against AI results (Fig. 7). Figure 8 shows that the prediction results of ANFIS method can fully match with CFD results and the randomization algorithm behind this training can improve the method for better tracking of each computing point and the amount of gas hold-up. We present CFD and AI data set in three dimensions to find pattern recognition of data set and compare CFD data and AI data set at the particular position of each computing element. The results show that the AI method can perfectly match gas fraction for each computing node, excluding only two local nodes. These regional nodes can be solved with the implementation of boundary conditions in the AI method.

**Figure 9:**
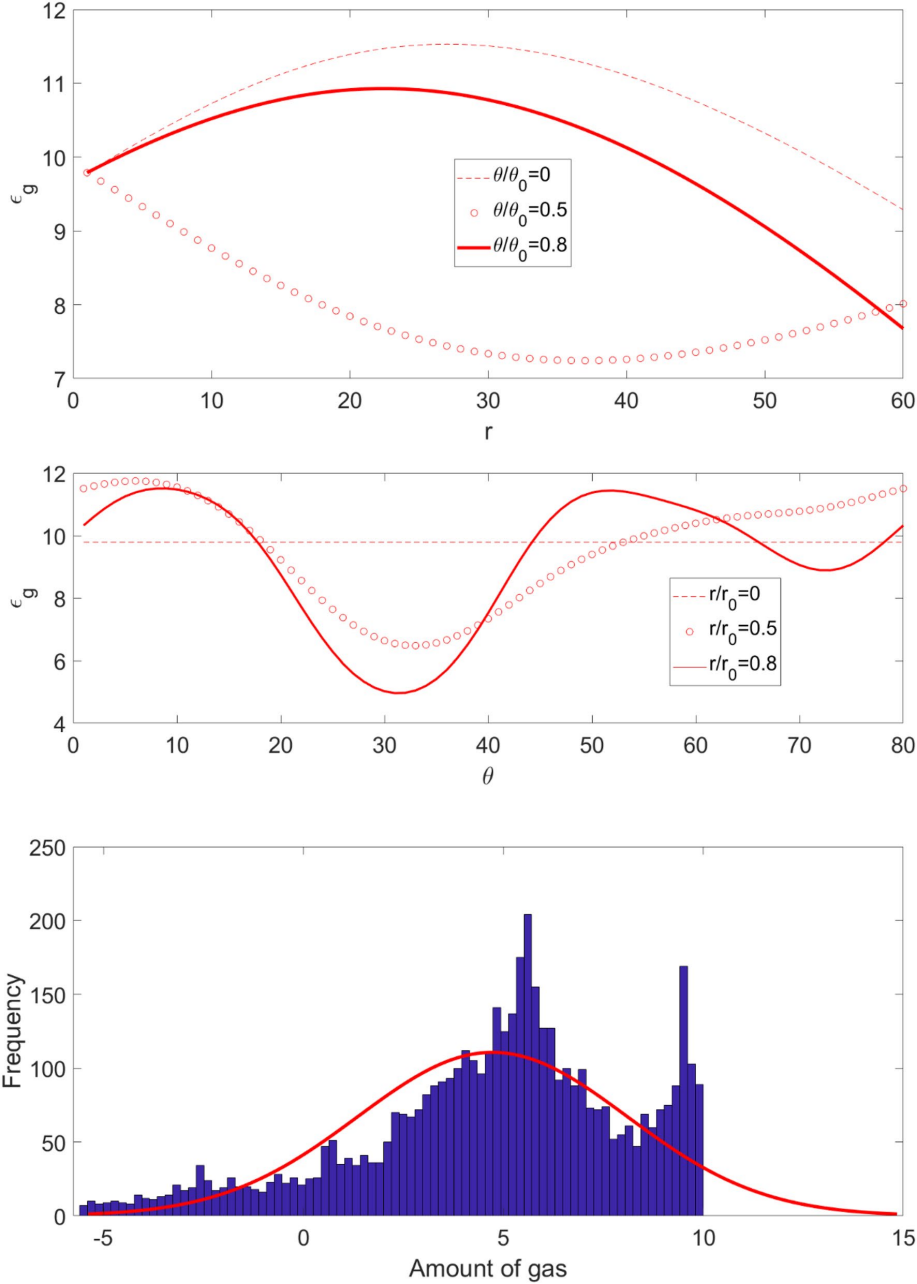
Gas distribution at different $\theta$ values for different $r$; Number of rules for training is two. Frequency of number of elements as a function of the amount of gas in the bubble column reactor.

Figure 9 shows the gas distribution for each element when $\theta$ is fixed in the polar coordinate and different *r* values. At *r* = 0 the amount of gas hold-up at all $\theta$ values are similar, while by increasing *r*, each element contains more gas. We understand the behavior of gas interaction by considering constant *r* value for different $\theta$ values. It also shows the gas distribution as a function of the $\theta$ value for three different *r* values. The figure shows that at $\theta = 34$ the minimum amount of gas accumulates at elements, while at 5 degree the amount of gas is maximum for different *r* values. Figure 9 shows the Frequency of number of elements as a function of the amount of gas in the reactor. The prediction tools cannot fully capture the amount of gas throughout the entire reactor. The results show that some of the elements contain negative gas hold-up.

**Figure 10:**
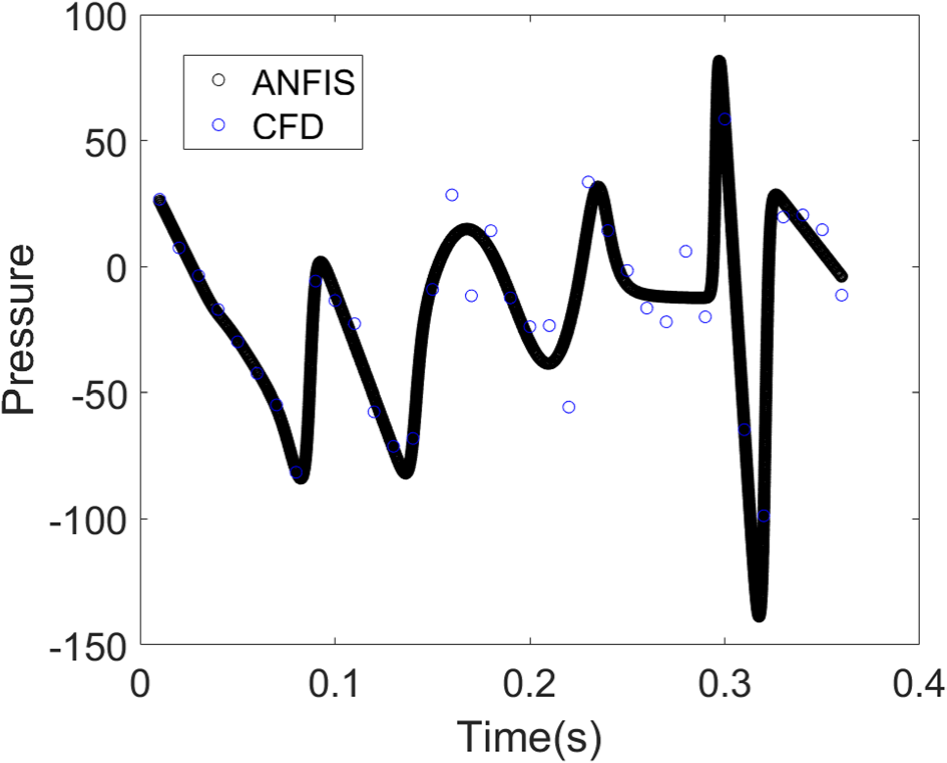
Time series calculation of pressure near the sparger orifice by CFD and ANFIS.

Figure 10 shows the time series for pressure distribution near the orifice by CFD and ANFIS. This time series contains 36 points while ANFIS method has $10^{5}$ computing points. The results reveal that ANFIS can fully cover the pattern of pressure distribution. The results also show that adding more AI nodes does not significantly change the computational time.

## Conclusions

The combination of neural cells relating to artificial learning method and fuzzy decision system can provide the data-driven learning method to learn turbulence multiphase flow calculated by *k −* *ε* model and single size Eulerian method. This learning method can use a selection of computing nodes to avoid training of the existing pattern of data in the bubble column reactor. The randomized structure can be used to select randomly 70% of overall data during learning. The rest of the non-training data is used for evaluation of this random selection of data. Additionally, the results of CFD are trained in the Cartesian coordinate and after training CFD data by ANFIS method, the turbulence multiphase flow is predicted in the polar coordinate. The turbulence flow characteristics and gas–liquid interaction data are trained and then tested for different $r$ and $\theta$. The results of artificial learning method show that we can predict turbulence flow characteristics and gas–liquid interaction data by combination of artificial intelligent algorithm and CFD. Computing nodes in AI method are not limited to the movement, and they can easily grow to provide high-resolution numerical results. Basically, as the ANFIS method is a data-driven model, the extrapolation of the process cannot be very accurate, particularly if the flow regime is changing in the domain, and more phenomena are happening. Basically, the physics behind the process must be calculated, and then the AI method can find other aspects of operations within the existing data-set. However, the mapping between different geometrical coordinates and the generation of the different mesh structures, and the number of elements is a possible task. In addition, results show that the AI model can estimate the value of gas fraction for each computing node at a particular location representing the correct pattern recognition process. This method can describe the refinement AI mesh structure for higher resolutions of gas fraction in each node.

## References

[1] Kantarci N, Borak F, Ulgen KO (2005). Bubble column reactors. Process. Biochem..

[2] Pourtousi M, Sahu J, Ganesan P (2014). Effect of interfacial forces and turbulence models on predicting flow pattern inside the bubble column. Chem. Eng. Process..

[3] Sarhan AR, Naser J, Brooks G (2018). CFD modeling of bubble column: influence of physico-chemical properties of the gas/liquid phases properties on bubble formation. Sep. Purif. Technol..

[4] Besagni G, Gallazzini L, Inzoli F (2019). On the scale-up criteria for bubble columns. Petroleum.

[5] Rollbusch P (2015). Bubble columns operated under industrially relevant conditions—current understanding of design parameters. Chem. Eng. Sci..

[6] Rezakazemi M, Shirazian S (2019). Gas–liquid phase recirculation in bubble column reactors: development of a hybrid model based on local CFD—adaptive neuro-fuzzy inference system (ANFIS). J. Non-Equilib. Thermodyn..

[7] Guo KY, Wang TF, Liu YF, Wang JF (2017). CFD–PBM simulations of a bubble column with different liquid properties. Chem. Eng. J..

[8] Sarhan AR, Naser J, Brooks G (2018). CFD model simulation of bubble surface area flux in flotation column reactor in presence of minerals. Int. J. Min. Sci. Technol..

[9] Yang Y, Chen YH, Wang YC, Li CH, Li L (2016). Modelling a combined method based on ANFIS and neural network improved by DE algorithm: a case study for short-term electricity demand forecasting. Appl. Soft Comput..

[10] McClure DD, Aboudha N, Kavanagh JM, Fletcher DF, Barton GW (2015). Mixing in bubble column reactors: experimental study and CFD modeling. Chem. Eng. J..

[11] Krishna R, Van Baten JM (2001). Scaling up bubble column reactors with the aid of CFD. Chem. Eng. Res. Des..

[12] Dhotre M, Ekambara K, Joshi J (2004). CFD simulation of sparger design and height to diameter ratio on gas hold-up profiles in bubble column reactors. Exp. Therm. Fluid Sci..

[13] Tabib MV, Roy SA, Joshi JB (2008). CFD simulation of bubble column—an analysis of interphase forces and turbulence models. Chem. Eng. J..

[14] Pourtousi M, Ganesan P, Sandaran SC, Sahu JN (2016). Effect of ring sparger diameters on hydrodynamics in bubble column: a numerical investigation. J. Taiwan Inst. Chem. Eng..

[15] Nguyen Q, Behroyan I, Rezakazemi M, Shirazian S (2020). Fluid velocity prediction inside bubble column reactor using ANFIS algorithm based on CFD input data. Arab. J. Sci. Eng..

[16] Pourtousi M, Ganesan P, Sahu J (2015). Effect of bubble diameter size on prediction of flow pattern in Euler–Euler simulation of homogeneous bubble column regime. Measurement.

[17] Zuo RG, Xiong YH, Wang J, Carranza EJM (2019). Deep learning and its application in geochemical mapping. Earth-Sci. Rev..

[18] Ghaedi AM, Vafaei A (2017). Applications of artificial neural networks for adsorption removal of dyes from aqueous solution: a review. Adv. Colloids Interface Sci..

[19] Lundervold AS, Lundervold A (2019). An overview of deep learning in medical imaging focusing on MRI. Z. Med. Phys..

[20] Azwadi CSN, Zeinali M, Safdari A, Kazemi A (2013). Adaptive-network-based fuzzy inference system analysis to predict the temperature and flow fields in a lid-driven cavity. Numer. Heat Transf. Part A Appl..

[21] Pourtousi M, Sahu J, Ganesan P, Shamshirband S, Redzwan G (2015). A combination of computational fluid dynamics (CFD) and adaptive neuro-fuzzy system (ANFIS) for prediction of the bubble column hydrodynamics. Powder Technol..

[22] Cao, Y., Babanezhad, M., Rezakazemi, M. & Shirazian, S. Prediction of fluid pattern in a shear flow on intelligent neural nodes using ANFIS and LBM. *Neural Computing and Applications*, 1–9 (2019).

[23] Tian, E., Babanezhad, M., Rezakazemi, M. & Shirazian, S. Simulation of a bubble-column reactor by three-dimensional CFD: multidimension-and function-adaptive network-based fuzzy inference system. *International Journal of Fuzzy Systems*, 1–14 (2019).

[24] Babanezhad M, Rezakazemi M, Hajilary N, Shirazian S (2019). Liquid-phase chemical reactors: development of 3D hybrid model based on CFD-adaptive network-based fuzzy inference system. Can. J. Chem. Eng..

[25] Xu LJ, Yuan BR, Ni HY, Chen CX (2013). Numerical simulation of bubble column flows in churn-turbulent regime: comparison of bubble size models. Ind. Eng. Chem. Res..

[26] Andrews MJ, Master BI (2005). Three-dimensional modeling of a Helixchanger((R)) heat exchanger using CFD. Heat Transf. Eng..

